# Asymmetric uncertainty regulation in human–artificial intelligence interaction

**DOI:** 10.3389/frai.2026.1847409

**Published:** 2026-09-01

**Authors:** Karim C. Abbaspour, Ario Saeid Vaghefi, Abbas Mirmashhouri, Nazanin Abbaspour, Markus Leippold, Mory Ghomshei

**Affiliations:** 1 2w2e Environmental Consulting GmbH, Dübendorf, Switzerland; 2 Environment and Climate, University of Zurich, Zürich, Switzerland; 3 WMO, Geneva, Switzerland; 4 Family Health Centers of San Diego, San Diego, CA, United States; 5 NutraLumina Integrative & Functional Nutrition, San Diego, CA, United States; 6 Tecto Energy Inc, Vancouver, BC, Canada; 7 Carnotech Energy Inc, Vancouver, BC, Canada

**Keywords:** asymmetric regulation, entropy dynamics, human-AI systems, non-vanishing entropy, stability margin, stochastic–affine systems

## Abstract

**Introduction:**

Human–AI interaction is commonly framed as a problem of uniformly minimizing uncertainty across the joint system. We challenge this assumption by proposing Asymmetric Uncertainty Regulation (AUR), a dynamical framework in which stable and adaptive collaboration requires directional rather than symmetric uncertainty regulation.

**Methods:**

Human–AI systems are modeled as coupled entropy dynamics. The artificial subsystem rapidly contracts predictive entropy through statistical inference and optimization, whereas the human subsystem maintains bounded but persistently non-zero entropy. System stability is characterized by a positive stability margin that prevents both symmetric entropy collapse and runaway instability. The framework is conceptually operationalized through illustrative applications in nutritional counseling, clinical diagnosis under novelty, and criminal investigation.

**Results:**

The model yields empirically testable signatures, including pronounced timescale separation between AI and human entropy trajectories, a non-zero human entropy plateau, and bounded fluctuations whose variance increases near the stability boundary. Systems that enforce symmetric entropy minimization are predicted to become rigid or fragile under distributional shift. By contrast, systems operating within the AUR regime are predicted to maintain predictive reliability while preserving exploratory variability, contextual adaptation, and value-sensitive human judgment.

**Discussion:**

AUR reframes uncertainty as a structured resource rather than a deficiency to be uniformly eliminated. The illustrative applications suggest how reliable AI prediction can be combined with human flexibility and normative judgment, although they constitute conceptual operationalizations rather than empirical validations. By preserving an asymmetry between AI certainty and bounded human uncertainty, AUR offers a principled foundation for designing human–AI systems that augment rather than erode human agency.

## 1 Introduction

A longstanding conceptual tension contrasts causal determination with human freedom, often associating causality with artificial intelligence and uncertainty with human agency. A more precise distinction concerns not causality itself, but how uncertainty evolves under the internal dynamics of interacting systems. Both human and artificial systems operate causally yet differ substantially in their uncertainty generation, regulation, and stabilization.

Human decision environments can be represented along two interacting dimensions: a causal dimension governed by relatively stable physical laws, institutional rules, and statistical relationships, and a human agency dimension in which creativity, interpretation, preferences, and contextual reasoning introduce forms of uncertainty not fully captured by predictive statistical models (Giddens, 1984). Behavioral economics has long documented systematic departures from classical rational choice through bounded rationality, heuristics, framing effects, context dependence, and attentional constraints (Simon, 1979; Kahneman and Tversky, 1979; Gigerenzer and Gaissmaier, 2011; Thaler, 2016; Gabaix, 2018; Kahneman et al., 2021; Bordalo et al., 2022). These approaches primarily characterize how human decisions deviate from idealized rational models. The present framework addresses a complementary question: what uncertainty-regulation architecture permits stable and adaptive interaction when human decision processes exhibiting bounded rationality, contextual sensitivity, and exploratory variability are coupled with artificial intelligence systems. AUR therefore does not seek to replace behavioral economics, but instead provides a dynamical systems perspective on how human cognitive variability can be preserved within human–AI interaction.

This distinction suggests complementary functional roles. Artificial intelligence excels at predictive inference, pattern recognition, and optimization, thereby contracting predictive uncertainty. Human agents contribute interpretive flexibility, exploratory variability, and value-driven reasoning that often extend beyond existing predictive structures. Effective hybrid systems therefore depend on asymmetric rather than uniform uncertainty dynamics. Importantly, improved alignment between human interpretation and AI prediction does not necessarily imply identical uncertainty regulation. Human–AI systems may converge toward shared objectives or improved predictive agreement while preserving complementary entropy dynamics that maintain contextual flexibility and adaptive exploration. Within AUR, alignment operates primarily at the level of objectives, predictions, or task performance, whereas asymmetry operates at the level of uncertainty regulation itself. If both subsystems contract uncertainty symmetrically, exploratory flexibility progressively diminishes, increasing vulnerability to novelty and distributional shift. Stable adaptation therefore benefits not merely from improved agreement, but from preserving complementary uncertainty dynamics alongside alignment.

Many contemporary approaches in machine learning, predictive modeling, and optimization seek to improve performance by reducing prediction error, predictive uncertainty, entropy, or expected surprise within artificial systems (Bishop, 2006; Friston, 2010; Murphy, 2012; Sutton and Barto, 2018; Ovadia et al., 2019; Steyvers and Kumar, 2024). When extended to coupled human–AI settings, these principles may implicitly encourage increasingly uniform uncertainty reduction across interacting subsystems through calibration, alignment, and convergence toward shared predictive states. At the same time, a substantial literature has examined complementary issues including automation bias, algorithm aversion and appreciation, trust calibration, appropriate reliance, human oversight, uncertainty communication, and complementary decision-making (Lee and See, 2004; Dzindolet et al., 2003; Parasuraman and Manzey, 2010; Dietvorst et al., 2015; Logg et al., 2019). Rather than replacing these perspectives, AUR provides a dynamical systems framework for analyzing how uncertainty itself evolves within coupled human–AI interactions. Specifically, AUR asks whether extending uncertainty minimization symmetrically across both subsystems preserves the exploratory flexibility, contextual adaptation, and resilience required under novelty and distributional shift.

The remainder of the paper develops this central principle from complementary perspectives: conceptually (Sections 2–3), mathematically (Section 4), empirically (Section 5), and operationally through design principles and applications (Section 6).

### 1.1 Formal definition of asymmetric uncertainty regulation (AUR)

We define AUR as a dynamical property of coupled human–AI systems in which the subsystems regulate uncertainty directionally. Let $H_{A}$ denote the uncertainty in the artificial subsystem and $H_{H}$ the uncertainty in the human subsystem, both quantified via Shannon entropy (see Table 1 for list of all symbols). A coupled system satisfies AUR when the following four structural conditions hold:

1. *Directional entropy contraction.* Artificial subsystems reduce predictive uncertainty more rapidly than human subsystems: $\lambda_{A}>\lambda_{H}$ , where $\lambda_{A}$ and $\lambda_{H}$ denote characteristic entropy contraction rates.
2. *Persistent human uncertainty*. Human uncertainty remains bounded and non-zero $\left(H_{H}>0\right)$ . Within AUR, this residual entropy emerges from competing cognitive, motivational, and contextual influences, including creativity, changing preferences, contextual reinterpretation, and value-sensitive reasoning, rather than from an explicit objective to maximize uncertainty. This bounded uncertainty supports exploratory capacity and adaptive flexibility.
3. *Preserved exploratory* var*iability*. Human subsystems retain sufficient exploratory variability to adapt to novel information, changing contexts, and evolving objectives.
4. *Dynamical stability*. The coupled uncertainty dynamics remain bounded, avoiding collapse into complete determinism or divergence into instability.

**Table 1:** Mathematical symbols and operational interpretations used in the AUR framework.

Symbol	Name	Units	Operational interpretation
t	Time	Time	Independent variable governing system evolution
$p_{A,i}(t)$	AI predictive distribution	Dimensionless (probability)	Probability assigned by the AI subsystem to outcome *i* at time *t*
$P_{H,i}(t)$	Human response distribution	Dimensionless (probability)	Probability assigned by the human subsystem to the same outcome i at time t
$H_{A}(t)$	AI entropy	Bits or nats	Shannon entropy of the AI predictive distribution, representing predictive uncertainty
$H_{H}(t)$	Human entropy	Bits or nats	Shannon entropy of the human response distribution, representing bounded exploratory uncertainty and contextual adaptation
H(t)	Entropy state vector	Bits or nats	Joint uncertainty state of the coupled human–AI system
$H_{\text{pred}}$	Predictive entropy component	Bits or nats	Predictive component associated with AI uncertainty contraction
$I_{\text{struct}}$	Structural information	Bits or nats	Structural informational divergence between human and AI probability distributions
M	Interaction matrix	1/time	Governs local contraction and cross-coupling dynamics
$\lambda_{A}$	AI contraction rate	1/time	Intrinsic rate of predictive uncertainty reduction in the AI subsystem
$\lambda_{H}$	Human relaxation rate	1/time	Intrinsic rate governing human uncertainty dynamics
γ	AI ← Human coupling	1/time	Influence of human entropy on AI entropy dynamics
δ	Human ← AI coupling	1/time	Influence of AI entropy on human entropy dynamics
$\overset{ˉ}{b}$	Drift vector	Bits/time	Mean exogenous entropy input vector
$b_{A}$	AI entropy injection	Bits/time	Simplifying modeling term for endogenous AI entropy injection, set to zero for predictive inference systems considered here
$\overset{ˉ}{\eta }$	Human entropy injection	Bits/time	Mean endogenous entropy input maintaining bounded human exploratory uncertainty and contextual adaptation
G	Diffusion matrix	(bits/time)^1/2^	Stochastic perturbations affecting entropy dynamics
$W_{t}$	Wiener process	Time^1/2^	Standard Brownian motion representing stochastic forcing
dH	Entropy increment	Bits or nats	Infinitesimal change in the entropy state vector
Σ	Covariance matrix	(bits)^2^	Stationary covariance of entropy trajectories
m	Stability margin	(1/time)^2^	Determinant-based measure classifying stable, critical, or unstable regimes
$H^{*}$	Equilibrium entropy	Bits or nats	Stationary mean entropy state toward which trajectories evolve
$D_{\mathrm{KL}}$	KL divergence	Bits or nats	Measure of structural divergence between human and AI probability distributions
α	Scaling coefficient	Dimensionless	Coefficient relating structural divergence to the informational decomposition
$I_{\mathrm{tot}}$	Total information	Bits or nats	Combined predictive and structural informational content
Σ(t)	Time-dependent covariance	(bits)^2^	Time-evolving covariance describing transient fluctuations
τ	Timescale asymmetry ratio	Dimensionless	Ratio of human to AI relaxation timescales, $\tau =\lambda_{H}/\lambda_{A}$
$R_{C}$	Normalized coupling strength	Dimensionless	Relative interaction strength, $R_{C}=\gamma \delta /(\lambda_{A}\lambda_{H})$

All entropy-related quantities are expressed using a common logarithmic base (bits or nats). Changing the logarithm base rescales entropy measures by a constant factor without altering the qualitative dynamics, stability conditions, or regime classification. The interpretations provided above are operational within the AUR framework and should not be construed as direct measurements of higher-level cognitive constructs such as creativity or agency.

Under AUR, predictive uncertainty contracts primarily within artificial subsystems while bounded uncertainty is preserved within human subsystems. This asymmetry enables predictive reliability and adaptive flexibility to coexist. Illustrative examples include AI-supported nutritional counseling (statistical optimization combined with clinician judgment of patient context), diagnosis under distributional shift, and criminal investigations that pair computational narrowing of possibilities with human exploration of alternative hypotheses.

Recent empirical work on human–AI collaboration supports the value of calibrated complementarity, where improved coordination does not require identical uncertainty dynamics across human and artificial subsystems (Steyvers and Kumar, 2024; Steyvers et al., 2025). Following prior work defining free will as structured unpredictability (Ghomshei and Abbaspour, 2026), we interpret the maintenance of bounded non-zero entropy under value-laden constraints as one dynamical condition supporting human agency, consistent with adaptive cognition (Mushtaq et al., 2011).

Subsequent sections formalize these principles as a coupled entropy-dynamical system, derive the stability conditions under which AUR can be maintained, characterize the geometry of the AUR regime, and examine empirical implications and falsifiable predictions.

## 2 Uncertainty as a structural feature of cognition (entropy formulation)

Uncertainty in human cognition is not merely an informational deficit relative to an underlying deterministic reality, but a structural feature of adaptive thought (Griffiths et al., 2010). Empirical research in cognitive science demonstrates systematic violations of classical probability theory, including order effects, context-dependent preference reversals, and non-commutative belief updating (Tversky and Kahneman, 1981; Wang et al., 2013; Trueblood and Busemeyer, 2011). These phenomena illustrate that human decision making depends on contextual framing, preferences, values, and other cognitive influences beyond purely probabilistic inference. Rather than serving as direct evidence for entropy dynamics, they motivate treating human uncertainty as an evolving consequence of multiple interacting processes rather than as a quantity that is uniformly minimized.

From an entropy perspective, human cognitive states are not fixed probability distributions awaiting measurement. Instead, the act of measurement or decision itself modifies entropy. Let $H_{H}$ denote the Shannon entropy associated with a human agent’s response distribution. This entropy is dynamically shaped by contextual framing, evolving goals, and interpretive processes.

### 2.1 Asymmetry between human and artificial uncertainty dynamics

In human–AI systems, uncertainty is regulated differently across the two subsystems, a central premise of the Asymmetric Uncertainty Regulation (AUR) framework. Unlike humans, whose uncertainty often evolves through open-ended reinterpretation and context-dependent belief revision, contemporary predictive AI systems are primarily designed to iteratively reduce predictive uncertainty during inference. Although the internal representations of modern AI systems, particularly large language models, are high-dimensional and need not correspond to well-defined hypothesis spaces, their operational behavior during inference is predominantly entropy-contractive. Through statistical inference and optimization, these systems typically reduce both epistemic and aleatoric uncertainty over the timescales relevant to human–AI interaction, although transient uncertainty expansion may occur during distributional shift, active exploration, or online adaptation (Hüllermeier and Waegeman, 2021). The AUR framework relies on this operational property rather than on any specific assumptions regarding the internal organization of AI representations.

The present framework is primarily intended for predictive AI systems that generate evolving probability distributions over task-relevant outcomes, including machine learning models, probabilistic forecasting systems, reinforcement learning architectures, and large language models operating in inference mode. Purely deterministic rule-based systems may be regarded as a limiting case with comparatively constrained entropy dynamics, while extending AUR to hybrid symbolic–statistical systems remains an important direction for future research.

Human uncertainty, by contrast, exhibits greater flexibility and context sensitivity. Entropy can increase through reinterpretation or exploration and decrease through commitment, without necessarily converging to zero. This non-monotonic, history-dependent behavior aligns with findings from quantum cognition models, which provide a formal language for contextuality and order effects in decision-making (Wang et al., 2013; Pothos and Busemeyer, 2013). In such frameworks, the entropy of a cognitive state depends on the order of measurements and the interaction history. Alignment operates at the level of goals/predictions; AUR asymmetry operates at the level of uncertainty dynamics.

Classical probabilistic models that incorporate sequential context-dependent updating can account for many observed order effects. Quantum probability provides an alternative formalism that has also been proposed to model such phenomena, although the present framework does not rely on a Hilbert-space representation or quantum ontology. The AUR framework is agnostic with respect to the particular mathematical formalism used to model human belief updating.

The resulting picture motivates modeling human–AI interaction as a coupled dynamical system in which the two subsystems exert cross-regulatory effects on each other’s entropy trajectories. Exogenous inputs and internal human processes prevent the complete collapse of human uncertainty, preserving the exploratory flexibility that is central to AUR. Section 3 formalizes these ideas by abstracting away from microscopic mechanisms and defining uncertainty regulation directly at the level of coupled entropy dynamics.

## 3 Mathematical formalization of asymmetric uncertainty regulation (stochastic–affine form with stability margin)

We adopt an information-theoretic formulation in which subsystem uncertainty, quantified by Shannon entropy, is the primary object of analysis. While earlier sections used operator-based representations for illustrative purposes, the structural definition of AUR depends only on time-dependent probability distributions over task-relevant outcomes. Open-system dynamics provide one natural mechanism for generating such distributions, but the AUR conditions themselves are defined at the distributional level.

Consider a coupled human–AI system composed of two subsystems:

- (A): the artificial (AI) subsystem,
- (H): the human subsystem.

Each subsystem maintains a time-dependent probability distribution over task-relevant outcomes.

### 3.1 Entropic uncertainty functional

Let $p_{A}^{\left(j\right)}(t)$ and $p_{H}^{\left(j\right)}(t)$ denote the AI predictive distribution and the human response distribution at time (t), respectively. The uncertainty of each subsystem is given by its Shannon entropy (the base of the logarithm may be 2 or (e); the choice does not affect comparative dynamics), (Equations 1–3). For finite discrete outcome spaces, Shannon entropy is bounded between zero and the logarithm of the number of admissible outcomes. The present framework focuses on the evolution of entropy within the interior of this bounded domain and does not explicitly model saturation at the upper boundary.

$$\;\;H_{A}(t)=-\sum_{j}p_{A}^{\left(j\right)}(t)\log p_{A}^{\left(j\right)}(t),$$ (1)

$$\;H_{H}(t)=-\sum_{j}p_{H}^{\left(j\right)}(t)\log p_{H}^{\left(j\right)}(t).\;\;$$ (2)

We collect the two entropies into the state vector

$$\begin{array}{cccc} & H(t)=(\begin{array}{c} H_{A}(t)\\ H_{H}(t) \end{array}). & & \end{array}$$ (3)

These scalar functionals summarize the magnitude of uncertainty without uniquely determining the underlying distributional microstates.

Within AUR, human entropy $\left(H_{H}\right)$ does not represent arbitrary behavioral noise, indecision, or incompetence. Shannon entropy is used operationally to characterize preserved exploratory variability within constrained human decision processes. It therefore reflects a maintained capacity for reinterpretation, contextual adaptation, exploration of alternatives, and flexible response generation.

Importantly, AUR does not assume that higher human entropy is always better. Extremely high entropy can impair coherent action, while entropy collapse can reduce adaptive flexibility (reflecting cognitive noise or confusion). The framework instead posits that effective human–AI interaction maintains bounded non-zero human entropy within an intermediate adaptive regime.

This bounded uncertainty is proposed as a necessary, but not sufficient, dynamical condition for higher-order functions such as creativity, agency, and value-sensitive reasoning to remain operational. Shannon entropy itself serves only as an operational descriptor of exploratory variability; these cognitive capacities require, but are not reducible to, bounded non-zero uncertainty. Preserved human entropy should therefore be interpreted as one supporting dynamical condition for adaptive cognition, not as a direct quantitative measure of cognitive quality or creativity.

### 3.2 Stochastic–affine coupled entropy dynamics

The stochastic–affine formulation introduced below is a minimal phenomenological model. It is not derived from a specific microscopic cognitive or algorithmic process; rather, it captures the essential structural features required by AUR: stronger intrinsic contraction in the AI subsystem, weaker contraction in the human subsystem, bidirectional coupling, and persistent exogenous entropy injection into the human subsystem. This level of abstraction enables tractable stability analysis while remaining agnostic to implementation details.

The joint entropy dynamics are modeled by the linear stochastic differential Equation 4:

$$\begin{array}{cccc} & dH(t)=(MH(t)+\overset{ˉ}{b})\mathrm{dt}+G(H(t))dW(t), & & \end{array}$$ (4)

Where the drift matrix (M) and the constant vector $\overset{ˉ}{b}$ are given by Equation 5:

$$\begin{array}{cccc} & M=(\begin{array}{cc} -\lambda_{A} & \gamma \\ \delta & -\lambda_{H} \end{array}),\overset{ˉ}{b}=(\begin{array}{c} b_{A}\\ \overset{ˉ}{\eta } \end{array}), & & \end{array}$$ (5)

Where $\lambda_{A}$ is intrinsic contraction rate of AI entropy, $\lambda_{H}$ is intrinsic contraction rate of human entropy ( $\lambda_{A}>\lambda_{H}\geq 0)$ , γ ( ≥0) is coupling strength from $H_{H}\;$ to $H_{A}$ , δ ( ≥0) is coupling strength from $H_{A}$ to $H_{H}$ , and G(H(t)) is a state-dependent diffusion matrix, which is a stochastic perturbations in entropy dynamics modulated near boundaries. We set $b_{A}=0$ , a simplifying assumption for predictive AI systems operating primarily in inference mode, while $\overset{ˉ}{\eta }>0$ represents the aggregate effect of persistent influences that introduce variability into the human subsystem, including creativity, contextual reinterpretation, evolving preferences, value-sensitive reasoning, and other informational and non-informational sources of utility not explicitly represented within the contractive dynamics (Hemmatian et al., 2024).

Beyond their mathematical role, the coupling parameters also admit a natural interpretation in human–AI interaction. The parameter ( δ ) represents the influence of AI outputs on human uncertainty, whereas ( γ ) captures the influence of human feedback, oversight, or correction on AI behavior. Appropriate values of these parameters are expected to depend on the application domain. High-risk settings, such as healthcare or aviation, may warrant stronger mechanisms for human oversight, whereas lower-risk or highly structured tasks may permit greater reliance on AI recommendations. This interpretation connects the AUR framework to the broader literature on calibrated trust, appropriate reliance, and complementary human–AI decision-making.

To ensure trajectories remain confined to the physically admissible state space ${\mathbb{R}}_{+}^{2}$ , the diffusion term G(H(t)) is specified as a state-dependent process whose noise amplitude scales with subsystem entropy (e.g., $G_{i}\propto \sqrt{H_{i}}$ ), analogous to a multidimensional Cox–Ingersoll–Ross construction. Under this formulation, stochastic fluctuations diminish naturally as a subsystem approaches absolute certainty $\left(H_{i}0\right)$ , preventing unphysical excursions beyond the entropy boundary. Importantly, because this modification acts only in the vicinity of the coordinate axes, the linear drift structure M and the determinant-based stability condition (m>0) remain unchanged as local asymptotic characterizations near the operating equilibrium $H^{*}$ . The present formulation does not explicitly incorporate upper entropy saturation associated with finite hypothesis spaces. Such saturation could be represented through nonlinear drift corrections or bounded-state stochastic processes. The current affine approximation assumes operation sufficiently far from these upper boundaries so that local linearization provides an adequate phenomenological description of the coupled dynamics. Accordingly, the stability analysis, phase-plane structure, and regime classifications presented in Section 4 are understood to hold within this structurally protected interior region.

When G(H(t))=0 , the system reduces to the deterministic affine dynamics expressed in Equation 6:

$$\begin{array}{cccc} & \frac{d}{\mathrm{dt}}E[H(t)]=ME[H(t)]+\overset{ˉ}{b}. & & \end{array}$$ (6)

Because Shannon entropy is strictly non-negative, the stochastic–affine formulation is interpreted as a local phenomenological approximation valid in the interior of the domain where $H_{A}(t)>0$ and $H_{H}(t)>0$ . A local Gaussian approximation may be used to describe sufficiently small fluctuations around a positive operating point and are not intended to capture boundary behavior near zero, where positivity constraints become dominant. All stability results, phase-plane analyses, and regime maps in Section 4 are therefore understood to apply within this interior regime. The stationary solutions and covariance structure characterize local stability properties rather than global guarantees over the entire entropy domain. Extensions incorporating reflecting boundaries, state-dependent diffusion, or nonlinear positivity-preserving dynamics remain important directions for future work.

### 3.3 Stability analysis and stability margin

The equilibrium mean entropy vector is Equation 7:

$$\begin{array}{cccc} & H^{*}=-M^{-1}\overset{ˉ}{b}, & & \end{array}$$ (7)

Which yields the explicit expressions in Equations 8:

$$\begin{array}{cccc} & H_{A}^{*}=\frac{\gamma \overset{ˉ}{\eta }}{m},\;\;\;\;H_{H}^{*}=\frac{\lambda_{A}\overset{ˉ}{\eta }}{m}, & & \end{array}$$ (8)

Where the stability margin is defined as in Equation 9:

$$\begin{array}{cccc} & m=\lambda_{A}\lambda_{H}-\gamma \delta . & & \end{array}$$ (9)

The eigenvalues of (M) have negative real parts if and only if tr(M)<0 and det(M)>0 . The trace condition $\mathrm{tr}(M)=-(\lambda_{A}+\lambda_{H})<0$ is automatically satisfied for positive contraction rates. The determinant condition simplifies to Equation 10:

$$\begin{array}{cccc} & m=\lambda_{A}\lambda_{H}-\gamma \delta >0. & & \end{array}$$ (10)

Thus m>0 is the central structural requirement for AUR. It guarantees:

- bounded entropy dynamics ( m>0 ),
- near-critical but responsive operation as $m\to 0^{+}$ , instability (runaway amplification) when m≤0 .

Strong coupling ( γ,δ large) is compatible with AUR provided the intrinsic contraction rates scale sufficiently to keep m>0 .

### 3.4 Emergent stationary human entropy

From Equation 8, the stationary human entropy is

$$\begin{array}{cccc} & H_{H}^{*}=\frac{\lambda_{A}\overset{ˉ}{\eta }}{m}, & & \end{array}$$ (11)

Where $m=\lambda_{A}\lambda_{H}-\gamma \delta >0$ is the stability margin.

Equation 11 shows that the stationary human entropy is jointly determined by the intrinsic contraction dynamics of the coupled system and the persistent exogenous entropy injection represented by $\overset{ˉ}{\eta }$ . Within the AUR framework, this positive stationary entropy should not be interpreted as a cognitive setpoint that human agents actively seek to maintain. Rather, it emerges as the equilibrium consequence of competing processes acting on the human subsystem.

The parameter $\overset{ˉ}{\eta }$ phenomenologically represents the aggregate influence of processes that continually introduce variability into human cognition, including creativity, contextual reinterpretation, evolving preferences, value-sensitive reasoning, and other informational or non-informational sources of utility that are not explicitly modeled within the contractive dynamics (Hemmatian et al., 2024). These competing influences counterbalance entropy contraction, preventing complete collapse of human exploratory variability while remaining consistent with bounded system stability.

If preservation of a minimum level of exploratory capacity is considered desirable for a particular human–AI application, the model implies the design condition Equation 12:

$$\begin{array}{cccc} & \overset{ˉ}{\eta }\geq \frac{m}{\lambda_{A}}H_{\min}, & & \end{array}$$ (12)

Where $H_{\min}>0$ denotes the minimum stationary entropy required for effective contextual adaptation and hypothesis exploration. This inequality should therefore be interpreted as a design or operating criterion rather than as evidence that humans consciously regulate uncertainty toward a fixed target.

Consequently, the positive stationary entropy predicted by the model is an emergent property of the coupled dynamics rather than an optimization objective. In the absence of persistent competing influences ( $\overset{ˉ}{\eta }\to 0$ ), the stationary solution approaches zero, whereas increasing contextual, motivational, or interpretive inputs raises the equilibrium entropy while remaining bounded by the stability condition m>0 . Within AUR, the resulting residual uncertainty provides the dynamical substrate that may support exploratory flexibility, reinterpretation, and adaptive reasoning, without implying that uncertainty itself is intrinsically beneficial or explicitly optimized.

### 3.5 Relation to the extended informational decomposition

Following the informational decomposition introduced in our previous work (Ghomshei and Abbaspour, 2026), total informational content is expressed as Equation 13:

$$I=H_{\text{pred}}+\alpha I_{\text{struct}},$$ (13)

Where $H_{\text{pred}}=H_{A}$ is the predictive entropy governed by the stochastic–affine dynamics. Here α>0 is a positive scaling coefficient that converts the Kullback–Leibler (Kullback and Leibler, 1951) divergence into the same entropy units used throughout the model, and

$$I_{\text{struct}}=D_{\mathrm{KL}}(p_{H}∥p_{A}),$$ (14)

Quantifies the structured informational divergence between the human response distribution $p_{H}$ and the AI predictive distribution $p_{A}$ .The distinction can be made concrete with a simple worked example. For illustration, let the AI distribution over four candidate actions be $p_{A}=(0.55,0.25,0.15,0.05)$ and the human distribution be $p_{H}=(0.40,0.30,0.20,0.10)$ . The corresponding entropies are $H_{A}\approx 1.58$ bits and $H_{H}\approx 1.85$ bits, while the structural divergence is $I_{\text{struct}}=D_{\mathrm{KL}}(p_{H}∥p_{A})\approx 0.13$ bits. This quantity cannot be recovered from the two scalar entropies alone, because it depends on the full distributions.

Importantly, $I_{\text{struct}}$ is treated as a derived informational observable rather than an additional dynamical state variable governed by the interaction matrix M . It is computed directly from the underlying probability distributions at each time t ; although its evolution could be locally linearized and incorporated into an augmented state-space model, such an extension is not required for the determinant-based stability analysis presented here.

The extended informational decomposition distinguishes productive human agency from undirected variability. Elevated human entropy ( $H_{H}$ ) alone does not imply meaningful adaptation, since it may arise from unstructured noise, poor calibration, or confusion. Instead, $I_{\text{struct}}$ captures systematic, context-sensitive deviations of human judgment from AI predictions.

Within the stable AUR regime ( m>0 ), preservation of a positive stationary entropy ( $H_{H}^{*}>0$ ) permits a corresponding structural component to persist, supporting exploratory flexibility, hypothesis generation, contextual reinterpretation, and adaptive reasoning. However, $I_{\text{struct}}$ is a descriptive informational quantity rather than a measure of performance. Structural divergence may reflect expertise, creativity, or alternative value structures, but may equally arise from cognitive bias, misinformation, or poor calibration. Its functional significance therefore cannot be determined from information-theoretic quantities alone and must instead be evaluated using external performance measures.

### 3.6 Empirical and operational implications

The stochastic–affine model makes several testable predictions. First, AI entropy $H_{A}(t)$ can be computed directly from model predictive distributions, while human entropy $H_{H}(t)$ can be estimated from observed behavioral response distributions. The stability margin (m) itself can be inferred by fitting the drift parameters to time series of entropies.

In the stable AUR regime, the system admits a local stationary approximation in a neighborhood of the positive operating equilibrium $H^{*}$ . Linearizing the state-dependent diffusion term around this operating point yields an effective Ornstein–Uhlenbeck approximation with locally constant diffusion $G(H^{*})$ . Under this local approximation, the covariance matrix Σ satisfies the continuous-time Lyapunov Equation 15:

$$\begin{array}{cccc} & \mathrm{M\Sigma}+\Sigma M^{⊤}+GG^{⊤}=0 & & \end{array}$$ (15)

The Lyapunov covariance equation is derived from a local Ornstein–Uhlenbeck approximation obtained by evaluating the state-dependent diffusion at the positive operating equilibrium $H^{*}$ . This Gaussian approximation is valid only when the characteristic fluctuation scale satisfies $\sqrt{\Sigma_{\mathrm{ii}}}\ll H_{i}^{*}$ , such that probability mass near or beyond the entropy boundary is negligible. As the stability margin approaches zero and fluctuations increase, this condition may fail. In that regime, the positivity-preserving state-dependent diffusion must be analyzed directly, and the Ornstein–Uhlenbeck covariance should be interpreted only as a qualitative indicator of critical slowing and increasing sensitivity.

As $m\to 0^{+}$ , stationary variance increases and relaxation times lengthen, consistent with critical slowing dynamics observed near complex-system transitions (Scheffer et al., 2009), signaling heightened sensitivity. When m≤0 , the linear approximation predicts diverging variance.

Operationally, a well-regulated human–AI system should satisfy Equation 16:

$$\begin{array}{cccc} & m>0,\lambda_{A}\gg \lambda_{H},H_{H}^{*}>0. & & \end{array}$$ (16)

Violation of m>0 produces dynamical instability, while collapse of $H_{H}^{*}$ toward zero indicates undesired symmetric entropy contraction.

Taken together, these results formalize the conditions under which asymmetric uncertainty regulation produces stable, bounded, and creative human–AI interaction.

## 4 Dynamical structure and stability of asymmetric uncertainty regulation

Section 3 established the stochastic–affine model of coupled entropy dynamics. This section analyzes the geometric structure, stability regimes, and qualitative behavior of the system in the entropy phase space $\left(H_{A},H_{H}\right)$ .

### 4.1 Structural stability of AUR

Stable AUR requires that the interaction matrix M (Equation 5) satisfies tr(M)〈0,det(M)〉0, or equivalently, $m=\lambda_{A}\lambda_{H}-\gamma \delta >0.$

Under these conditions, entropy trajectories remain bounded and converge in distribution to the stationary stochastic regime centered on the equilibrium $H^{*}$ (Equation 8). A defining feature of this regime is directional entropy contraction: AI entropy ( $H_{A}$ ) contracts rapidly ( $\lambda_{A}\gg \lambda_{H}$ ), whereas human entropy ( $H_{H}$ ) evolves more slowly around a positive stationary level sustained by the exogenous entropy injection $\overset{ˉ}{\eta }>0$ .

### 4.2 Stationary stochastic regime

In the stable regime, entropy trajectories fluctuate around the equilibrium $H^{*}$ with bounded covariance determined by the Lyapunov equation (Equation 15). As the stability margin m approaches zero from above, relaxation slows and fluctuation amplitude increases, providing an early indicator of structural fragility (Scheffer et al., 2009).

### 4.3 Failure modes of symmetric uncertainty minimization

If the contraction rates become comparable ( $\lambda_{A}\approx \lambda_{H}$ ) and the human entropy injection vanishes ( $\overset{ˉ}{\eta }\to 0$ ), the system enters a symmetric uncertainty-minimization regime. In this limit, either both entropies collapse toward zero, eliminating exploratory capacity, or the stability margin becomes non-positive, leading to dynamical instability.

The directional timescale asymmetry assumed by AUR should be interpreted as a characteristic operating tendency rather than an instantaneous constraint. Temporary departures from $\lambda_{A}\gg \lambda_{H}$ may occur during adaptation to novel conditions without violating the framework, provided the long-term contractive structure is preserved.

### 4.4 Phase-plane geometry

**Figure 1:**
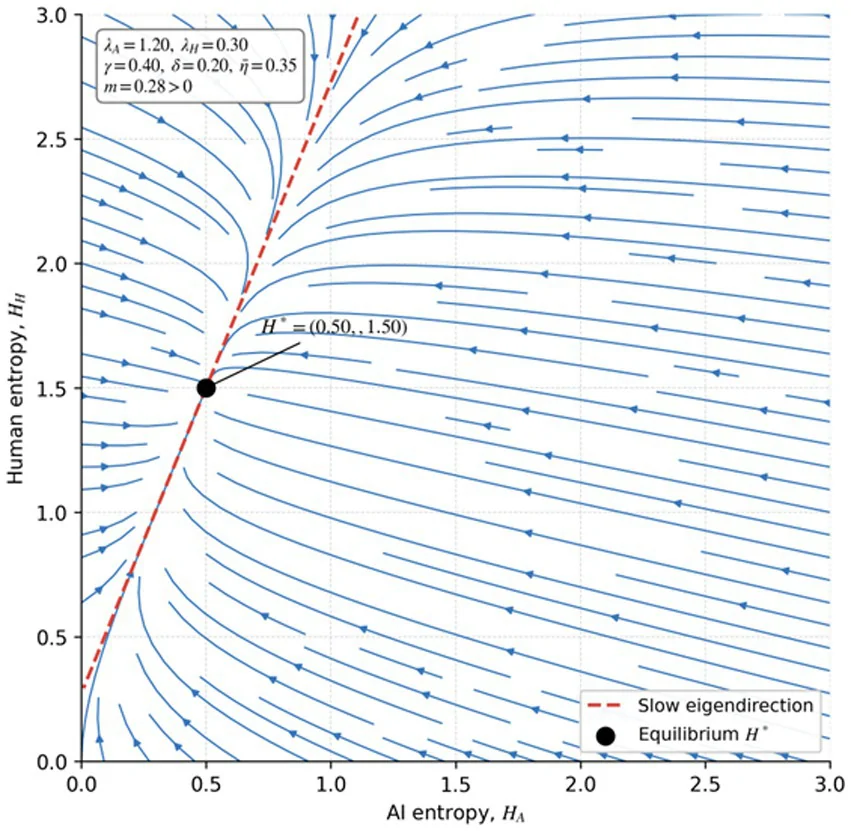
Deterministic phase portrait of the coupled entropy dynamics. Streamlines generated from $\dot{H}=\mathrm{MH}+b$ converge to the stable equilibrium $H^{*}=(0.50,1.50)$ . The dashed line indicates the calculated slow eigendirection. For the illustrated parameters, m=0.28>0 , and both eigenvalues are real and negative.

**Figure 2:**
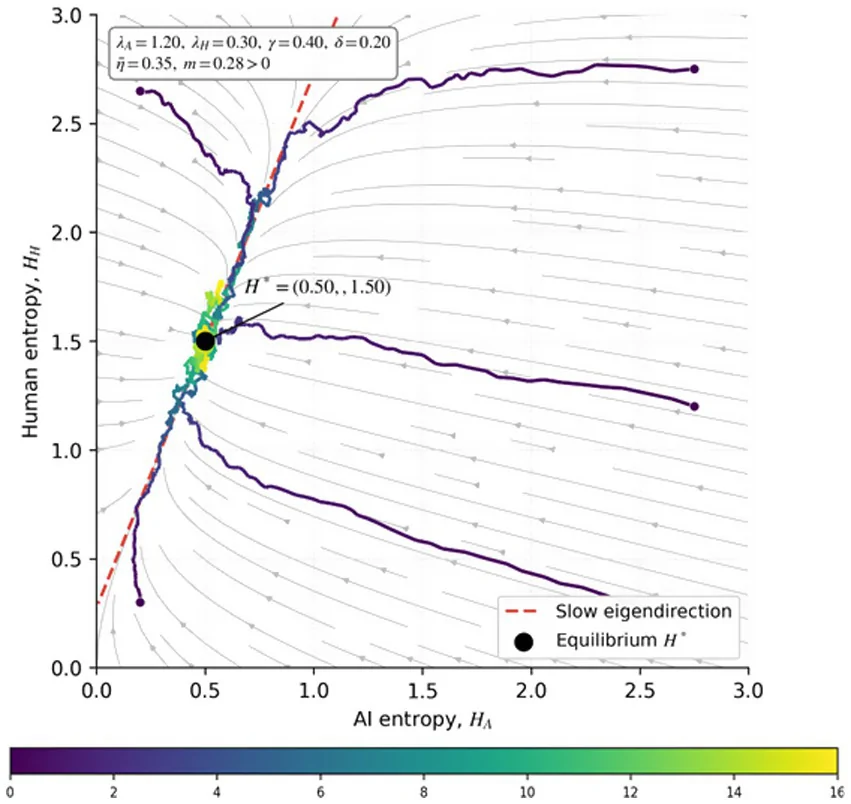
Representative stochastic trajectories in the entropy phase plane. Trajectories generated with positivity-preserving state-dependent diffusion initially contract rapidly in AI entropy and subsequently align with the calculated slow eigendirection before fluctuating near the stable equilibrium $H^{*}=(0.50,1.50)$ . Color indicates increasing time.

The deterministic drift field generated by M produces trajectories that converge toward the stationary point $H^{*}$ in the $\left(H_{A},H_{H}\right)$ phase plane (Figure 1). Because $\lambda_{A}\gg \lambda_{H}$ , convergence is anisotropic: trajectories rapidly approach the slow eigendirection before gradually relaxing to equilibrium (Figure 2). This separation of timescales is the geometric signature of asymmetric uncertainty regulation.

**Figure 3:**
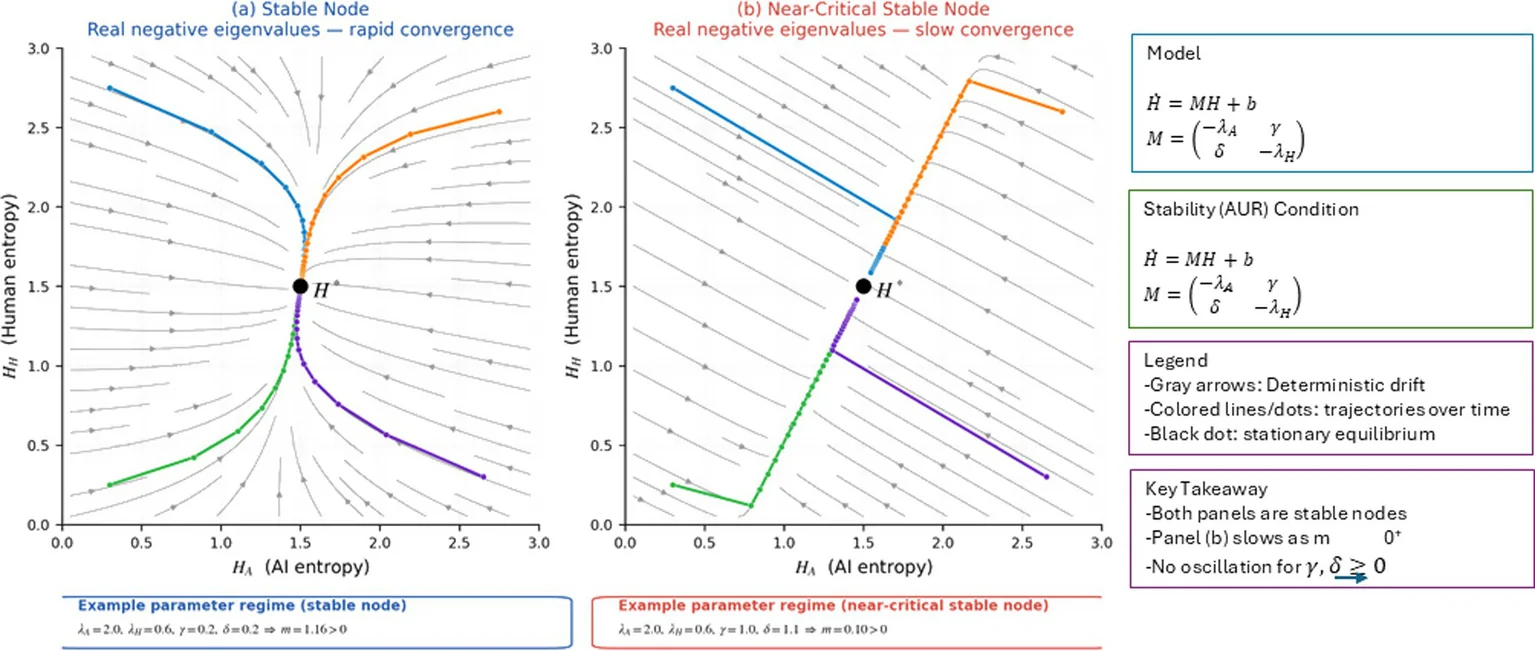
Stable-node convergence patterns in the entropy phase plane. **(a)** Monotonic convergence toward equilibrium. **(b)** Transient non-monotonic behavior of individual state variables, followed by convergence to the stable equilibrium $H^{*}$ . With nonnegative coupling parameters, oscillatory or spiral convergence cannot occur.

Under the assumed nonnegative bidirectional coupling parameters, the eigenvalues of *M* are always real. Therefore, whenever m > 0, the deterministic system converges to equilibrium as a stable node. Individual state variables may exhibit transient non-monotonic behavior, depending on the initial condition, but genuine oscillatory or spiral convergence cannot occur (Figure 3).

### 4.5 Structural origin of asymmetry

**Figure 4:**
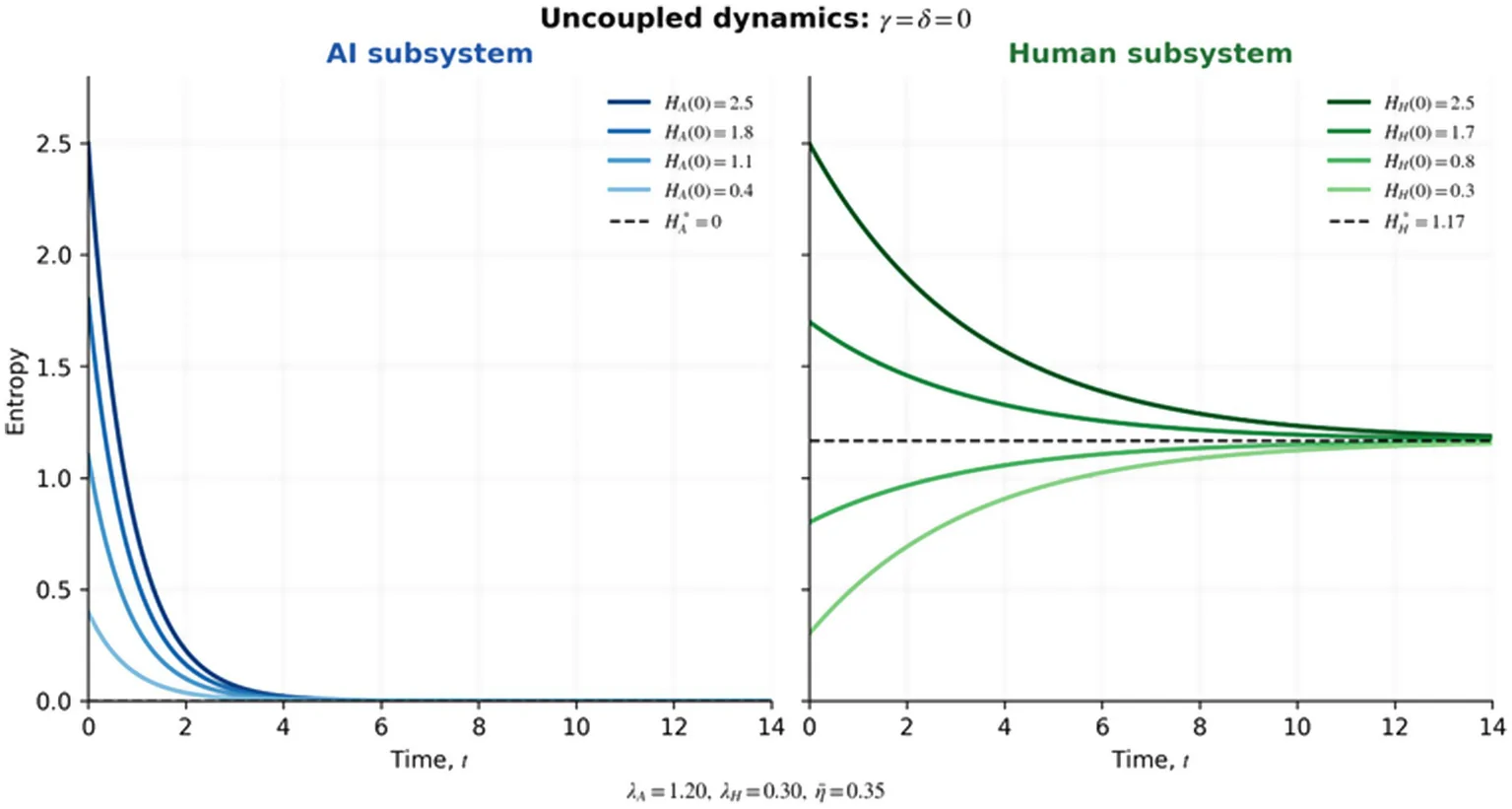
Uncoupled subsystem dynamics ( γ=δ=0 ). AI entropy rapidly converges to $H_{A}^{*}=0$ , whereas human entropy approaches the positive stationary level $H_{H}^{*}=\overset{ˉ}{\eta }/\lambda_{H}=1.17$ . Curves represent different initial conditions.

The AI and human subsystems exhibit fundamentally different intrinsic dynamics (Figure 4). Under the assumed contractive dynamics, the AI subsystem rapidly converges toward its operating level, whereas the human subsystem approaches a positive stationary entropy sustained by persistent exogenous entropy injection. When coupled, this intrinsic asymmetry is preserved, giving rise to a stable entropy manifold that maintains bounded human variability without destabilizing the overall system.

### 4.6 Regime structure and dimensionless parameters

The qualitative behavior of the coupled system is governed by two dimensionless parameters. The timescale asymmetry parameter is as in Equation 17:

$$\tau =\frac{\lambda_{H}}{\lambda_{A}},\;0<\tau \ll 1,$$ (17)

Which quantifies the separation of contraction rates between the subsystems. Smaller values of τ produce stronger anisotropy and a more pronounced slow manifold in the phase plane.

The normalized coupling strength is given by Equation 18:

$$R_{C}=\frac{\gamma \delta }{\lambda_{A}\lambda_{H}}<1,$$ (18)

Which measures the relative importance of cross-subsystem feedback. Stable AUR therefore requires $R_{C}<1$ .

### 4.7 Stability regimes

**Figure 5:**
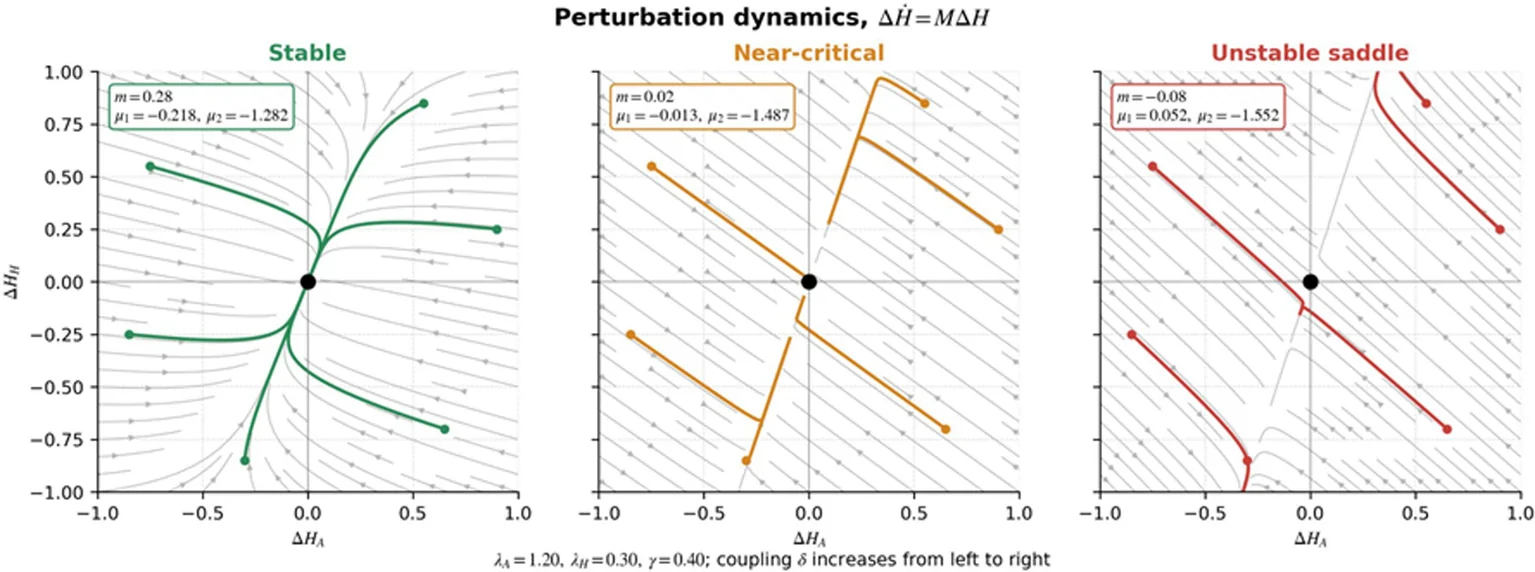
Stability regimes of the coupled system. Perturbation trajectories $\mathrm{\Delta H}=H-H^{*}$ converge rapidly for m=0.28 , converge slowly near the stability boundary for m=0.02 , and exhibit saddle instability for m=−0.08 . In the unstable regime, trajectories along the stable manifold approach the equilibrium, whereas trajectories with a component along the unstable eigendirection diverge.

The coupled system exhibits three qualitatively distinct regimes determined by the stability margin m (Figure 5). For m>0 , deterministic restoring forces dominate, producing bounded fluctuations around $H^{*}$ . As $m\to 0^{+}$ , restoring forces weaken, relaxation slows, and fluctuation amplitude increases. When m≤0 , stability is lost and coordinated regulation between the human and AI subsystems breaks down.

### 4.8 Regime map

**Figure 6:**
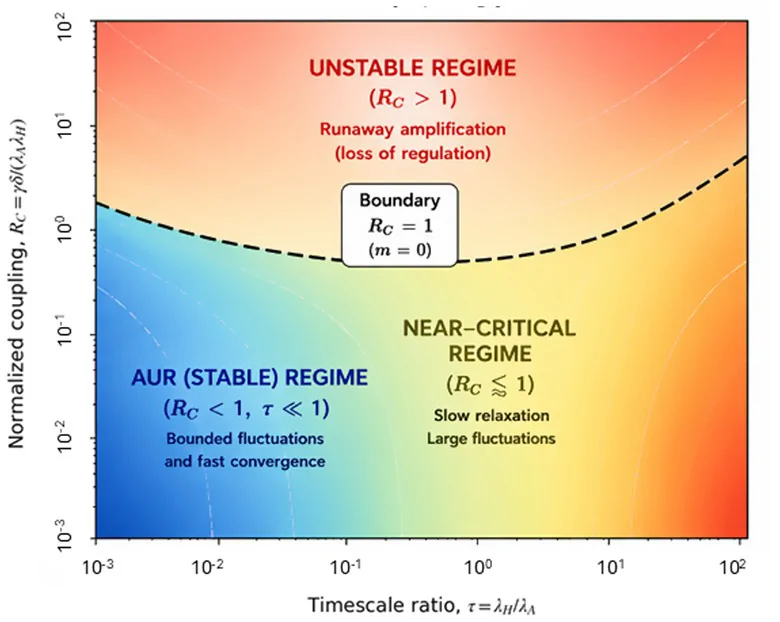
Regime map of coupled human–AI entropy dynamics. Stability is determined by normalized coupling $R_{C}$ : the system is stable for $R_{C}<1$ , approaches critical slowing as $R_{C}\to 1^{-}$ , and is unstable for $R_{C}>1$ . The timescale ratio τ characterizes the degree of subsystem asymmetry but does not independently determine stability. The point identifies the parameter regime used in Figures 1, 2.

The dimensionless parameters τ and $R_{C}$ define a low-dimensional regime map for human–AI interaction (Figure 6). For $\tau \ll 1,R_{C}<1,$ the system operates in the AUR regime, characterized by rapid AI entropy contraction, bounded human entropy, and anisotropic convergence. As $R_{C}\to 1$ or τ→1 , the system approaches a near-critical regime with slower relaxation and larger fluctuations. When $R_{C}\geq 1,$ or $\tau \approx 1,\overset{ˉ}{\eta }\to 0,$ the dynamics transition to instability or symmetric entropy collapse.

### 4.9 Informational interpretation

Within the extended informational decomposition (Section 3.5), the phase-plane dynamics admit a natural informational interpretation. The inward drift primarily reduces predictive entropy, $H_{\text{pred}}=H_{A},$ while the preserved human entropy floor supports the structural informational component, $I_{\text{struct}}=\alpha D_{\mathrm{KL}}(p_{H}∥p_{A}).$

Consequently, the geometric asymmetry of AUR provides the dynamical basis for maintaining structured informational divergence and exploratory variability.

## 5 Operationalization and illustrative applications of AUR

Sections 3 and 4 established AUR as a stochastic–affine entropy-dynamical regime characterized by rapid AI entropy contraction and bounded, persistently non-zero human entropy. This section outlines its empirical signatures, measurement protocol, and illustrative applications.

### 5.1 Observable signatures of AUR

The stochastic–affine model yields five directly testable signatures of stable AUR:

1. *Timescale separation:* AI entropy $H_{A}(t)$ decreases substantially faster than human entropy $H_{H}(t)$ , consistent with $\lambda_{A}\gg \lambda_{H}$ .
2. *Positive human entropy plateau:* Human entropy converges to a strictly positive stationary level $H_{H}^{*}>0$ , sustained by persistent exogenous entropy injection $\overset{ˉ}{\eta }>0$ .
3. *Bounded fluctuations:* Entropy trajectories exhibit stationary variance and finite autocorrelation determined by the Lyapunov covariance equation (Equation 15). Increasing variance and slowing relaxation indicate proximity to the critical boundary $m\to 0^{+}$ .
4. *Stability margin:* The interaction satisfies $m=\lambda_{A}\lambda_{H}-\gamma \delta >0,$ placing the system within the stable AUR region of the regime map (Figure 6).
5. *Outcome-dependent structural divergence:* Structural information $\left(I_{\text{struct}}\right)$ should be interpreted jointly with independent task-performance measures. Persistent divergence associated with improved adaptation, resilience to novelty, or enhanced decision quality is consistent with adaptive exploratory variability under AUR.

Collectively, these signatures distinguish asymmetric uncertainty regulation from symmetric entropy collapse and dynamical instability.

### 5.2 Measurement protocol

**Figure 7:**
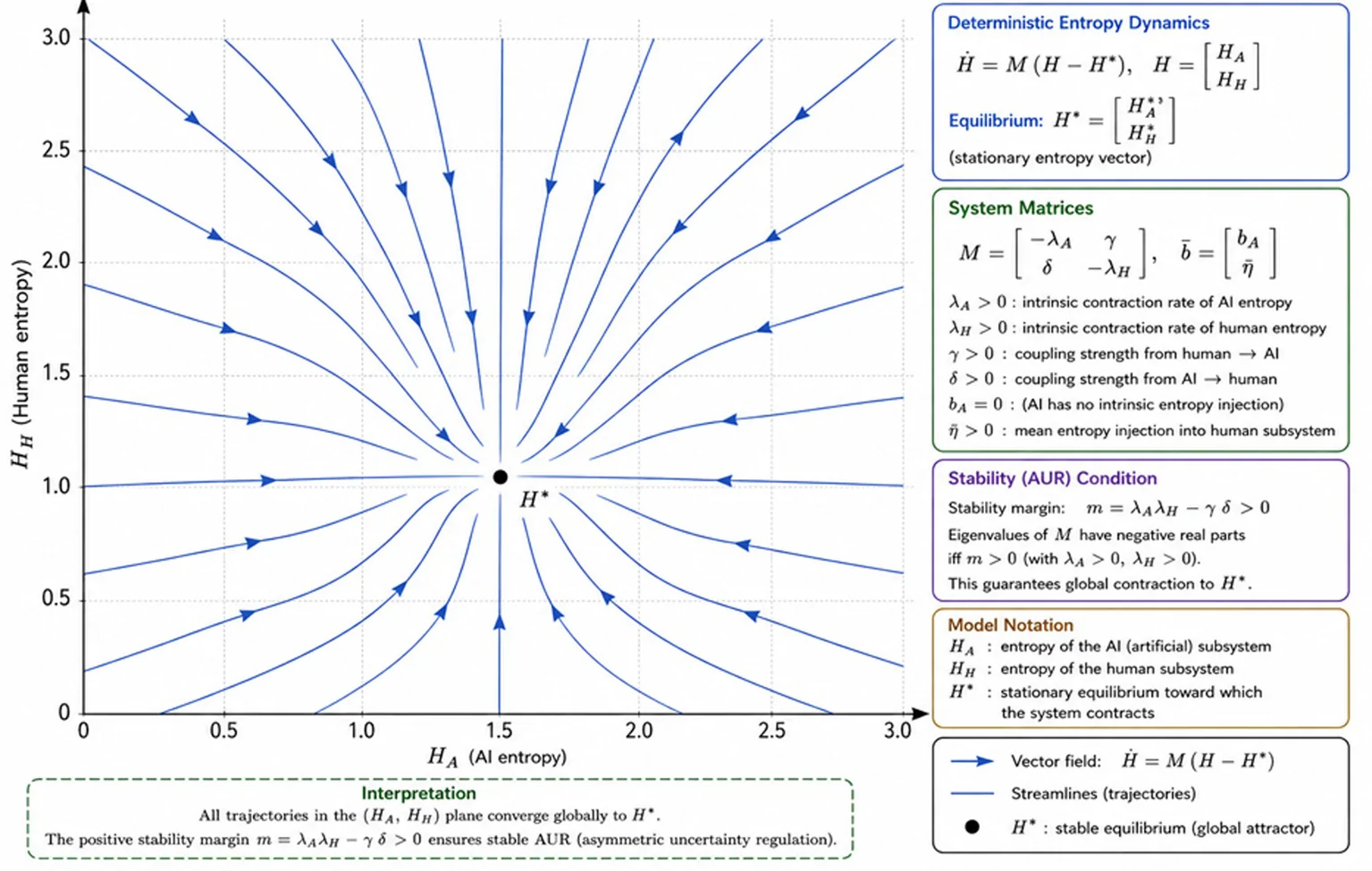
Conceptual protocol for estimating entropy trajectories in human–AI systems. AI entropy $H_{A}(t)$ is estimated from predictive probability distributions or confidence scores, whereas human entropy $H_{H}(t)$ is estimated from behavioral responses, inter-rater variability, or elicited subjective probabilities. The resulting trajectories provide operational observables for parameter estimation and evaluation of the AUR stability margin *m*.

Empirical evaluation of AUR proceeds by estimating entropy trajectories from observed data. AI entropy $H_{A}(t)$ is computed from probabilistic model outputs, whereas human entropy $H_{H}(t)$ is estimated from behavioral response distributions, including repeated choices, inter-rater variability, or elicited subjective probabilities (Figure 7).

Standard time-series methods can then estimate the model parameters $(\lambda_{A},\lambda_{H},\gamma ,\delta ,\overset{ˉ}{\eta })\;$ and compute the stability margin m . The protocol is applicable to existing datasets in clinical decision support, forecasting, and collaborative problem solving.

Entropy-based observables should be interpreted together with independent task-performance measures—including predictive accuracy, adaptation to distributional shift, recovery under novel conditions, calibration quality, and decision outcomes—to distinguish productive exploratory variability from unstructured noise or systematic bias.

The following examples are illustrative and are intended to demonstrate the operational interpretation of asymmetric uncertainty regulation in familiar human–AI decision environments.

### 5.3 Illustrative applications

The following examples illustrate how the AUR framework may be operationalized across different domains. The numerical trajectories are representative and heuristic rather than empirical, and are intended solely to demonstrate the qualitative behavior of the model. Empirical validation will require longitudinal human–AI interaction datasets and parameter estimation from observed entropy trajectories.

It should be noted that because $D_{\mathrm{KL}}(p_{H}\;\|p_{A})$ depends on the full underlying distributions and is not uniquely determined by the scalar entropy states $H_{A}$ and $H_{H}$ , the $I_{\text{struct}}$ values in the illustrative examples are stipulated for demonstration and are not outputs of the two-state dynamical model.

#### 5.3.1 Nutritional counseling (detailed example)

Predictive probability distributions over candidate dietary strategies (e.g., Mediterranean, low-carbohydrate, plant-forward, or DASH patterns) using biomarkers, clinical evidence, and population-level nutritional data. The human practitioner maintains a probability distribution over the same intervention space while incorporating contextual factors that are not fully represented by statistical optimization, including cultural preferences, lifestyle constraints, adherence feasibility, patient values, and motivation. Entropy is evaluated using Shannon entropy over these distributions at each counseling session.

To illustrate the resulting dynamics, we consider a representative parameter configuration within the stable AUR regime:

$$\lambda_{A}=1.20,\lambda_{H}=0.15,\gamma =0.10,\delta =0.02,\overset{ˉ}{\eta }=0.205.$$

This configuration yields a positive determinant stability margin,

$$m=\lambda_{A}\lambda_{H}-\gamma \delta =0.178>0,$$

satisfying Equation 10 and placing the system within the stable AUR regime. The relatively small AI-to-human coupling ( δ=0.02 ) limits excessive compression of human uncertainty, while positive human-to-AI coupling ( γ=0.10 ) allows contextual information to influence AI adaptation without destabilizing the coupled system. Representative entropy trajectories are shown in Table 2.

**Table 2:** Illustrative entropy evolution during nutritional counseling.

Session	$H_{A}$	$H_{H}$	$I_{\text{struct}}$
0	1.28	1.36	0.42
3	0.77	1.37	0.78
7	0.39	1.37	1.12

As patient-specific information accumulates, the AI predictive distribution becomes increasingly concentrated, producing rapid entropy contraction ( 1.28→0.39 ), whereas human entropy remains close to a positive stationary level ( $H_{H}\approx 1.37$ ) through persistent contextual information injection ( $\overset{ˉ}{\eta }=0.205$ ) and slower intrinsic contraction dynamics.

The maintained human entropy reflects preservation of interpretive capacity rather than passive uncertainty. Accordingly, structural information increases from ( $I_{\text{struct}}=0.42\;\;\text{to}\;1.12)$ , indicating that preserved human variability supports context-sensitive divergence as AI predictions become more concentrated. This residual uncertainty enables patient-specific adaptation, exploration of clinically relevant alternatives, and moderation of purely algorithmic recommendations, allowing the final nutritional strategy to emerge from statistical optimization refined by human clinical judgment.

#### 5.3.2 Novel pathogen response

The emergence of a novel pathogen provides a natural illustration of AUR under conditions of abrupt distributional shift and elevated predictive uncertainty. An AI epidemiological system generates probability distributions over transmission dynamics, mutation trajectories, and candidate intervention strategies using information derived from previously observed pathogens. Simultaneously, public health officials and clinical researchers maintain an independent hypothesis space informed by physiological principles, evolutionary mechanisms, clinical expertise, and institutional memory. Subsystem uncertainty is quantified using Shannon entropy over these predictive and decision distributions.

To illustrate these dynamics, we consider the representative parameter configuration: $\lambda_{A}=0.80,\lambda_{H}=0.30,\gamma =0.125,\delta =0.40,\overset{ˉ}{\eta }=0.273$ .

The corresponding stability margin is: $m=\lambda_{A}\lambda_{H}-\gamma \delta =0.240-0.05=0.190>0$ , which satisfies Equation 10 and places the system within the stable AUR regime. Representative entropy trajectories are summarized in Table 3.

**Table 3:** Illustrative entropy evolution during novel pathogen response.

Phase	$H_{A}$	$H_{H}$	$I_{\text{struct}}$
Baseline (Pre-Outbreak)	0.10	0.80	0.25
Novelty Exposure (T + 1)	0.35	1.79	1.45
Adaptive Convergence (T + 2)	0.18	1.15	0.82

Prior to pathogen emergence, the AI subsystem operates with relatively low predictive entropy ( $H_{A}=0.10$ ). Exposure to a previously unobserved pathogen produces a transient increase in predictive entropy (0.10 → 0.35) as the system encounters conditions outside its prior statistical experience. This temporary increase represents an out-of-equilibrium perturbation rather than a loss of dynamical stability, since the interaction matrix continues to satisfy m > 0.

The elevated predictive uncertainty propagates through the coupled system and is accompanied by expansion of human exploratory entropy (0.80 → 1.79) as clinicians and researchers broaden their hypothesis space to evaluate competing mechanisms and emerging evidence. Consistent with this behavior, the structural information component increases ( $I_{\text{struct}}:0.25\to 1.45$ ) indicating systematic divergence between human judgment and AI statistical predictions during adaptation rather than unstructured variability or random noise.

As empirical evidence accumulates, bidirectional coupling facilitates progressive adaptation. Human contextual interpretation contributes updated constraints through the human-to-AI coupling (*γ* = 0.125), while the AI subsystem progressively restores predictive concentration through its intrinsic contractive dynamics (λ_A_ = 0.80). The coupled system ultimately converges to a new stable operating regime while preserving the asymmetric uncertainty regulation characteristic of AUR.

#### 5.3.3 Criminal investigation

Complex criminal investigations require integration of heterogeneous information sources, including digital forensic evidence, financial transactions, geospatial histories, communication patterns, and witness testimony. Within this setting, an AI subsystem generates predictive probability distributions over investigative leads, suspect networks, and evidentiary relationships using statistical inference, anomaly detection, and pattern-recognition methods. Simultaneously, human investigators maintain an independent probability distribution over the investigative hypothesis space, incorporating contextual information that may not be fully represented within algorithmic optimization, including behavioral interpretation, situational context, motive plausibility, and evolving investigative priorities. Subsystem uncertainty is quantified using Shannon entropy over these predictive and investigative distributions.

To illustrate these dynamics, we consider the representative parameter configuration

$$\lambda_{A}=1.00,\lambda_{H}=0.20,\gamma =0.15,\delta =0.10,\overset{ˉ}{\eta }=0.17.$$

The corresponding stability margin is:

$$m=\lambda_{A}\lambda_{H}-\gamma \delta =0.200-0.015=0.185>0,$$

Which satisfies Equation 10 and places the system within the stable AUR regime. Under this parameterization, moderate bidirectional coupling allows AI predictions to guide investigative focus while enabling human contextual interpretation to provide adaptive constraints. Representative entropy trajectories are summarized in Table 4.

**Table 4:** Illustrative entropy evolution during criminal investigation.

Phase	$H_{A}$	$H_{H}$	$I_{\text{struct}}$
Initial Discovery	1.15	1.50	0.35
Evidentiary Narrowing	0.45	0.95	0.88
Contextual Pivot	0.72	1.12	1.34

During the initial discovery phase, both subsystems operate under elevated uncertainty as multiple competing hypotheses remain viable. As physical and digital evidence accumulates, AI predictive entropy contracts from *H_A_* = 1.15 to 0.45, consistent with its intrinsic contractive dynamics (λ_A_ = 1.00). Human entropy also decreases from *H_H_* = 1.50 to 0.95 as the investigative hypothesis space becomes progressively constrained by accumulating evidence.

Persistent contextual information injection (η̄ = 0.17), however, prevents complete collapse of human exploratory capacity. During the subsequent *Contextual Pivot* phase, reinterpretation of witness testimony or the emergence of conflicting evidence broadens the investigative hypothesis space, increasing human entropy from 0.95 to 1.12. At the same time, the structural information component increases from ( $I_{\text{struct}}:0.88\to 1.34$ ), indicating systematic divergence between human judgment and AI statistical predictions rather than unstructured variability.

Through bidirectional coupling (*γ* = 0.15), this contextual reinterpretation propagates back into the coupled system and is associated with a temporary increase in AI predictive entropy from 0.45 to 0.72, reflecting expansion of the algorithmic search space under revised informational constraints. As additional evidence accumulates, both subsystems progressively reconverge toward a stable operating regime while preserving the complementary roles of statistical inference and contextual interpretation.

This example illustrates how asymmetric uncertainty regulation supports adaptive decision making in adversarial, information-rich environments by balancing predictive concentration with maintained exploratory flexibility.

### 5.4 Failure regimes

The framework identifies three observable failure modes of human–AI interaction:

- *Symmetric collapse:* Both entropies converge toward zero, producing rigid, overconfident decisions that are brittle under distributional shift.
- *Coupling-induced instability:* Excessive cross-subsystem feedback drives *m* ≤ 0, leading to diverging fluctuations and loss of coordinated regulation.
- *Near-critical fragility:* The system remains formally stable *m* → 0^+^, but exhibits large variance, slow relaxation, and heightened sensitivity to perturbations.

These regimes define the empirical boundaries of effective asymmetric uncertainty regulation.

### 5.5 Influence decomposition

Complementary to the entropy dynamics, the relative influence of each subsystem on final decisions can be expressed through normalized influence weights $w_{A}$ and $w_{H}$ satisfying $w_{A}+w_{H}=1$ . A practical operationalization links influence to confidence measures as expressed is

$$w_{A}=\frac{c_{A}}{c_{A}+c_{H}},\;\;w_{H}=\frac{c_{H}}{c_{A}+c_{H}},$$ (19)

Where $c_{A}$ and $c_{H}$ are non-negative confidence indices appropriate to their respective subsystems.

For AI systems, confidence may be quantified using measures such as inverse predictive entropy, maximum predicted probability, or calibrated confidence scores derived from the model’s predictive distribution. For human decision makers, confidence is generally measured using different methodologies, including elicited confidence ratings (e.g., calibrated subjective probability judgments) or, in settings involving multiple decision makers, inter-rater agreement measures such as Cohen’s κ or Krippendorff’s α . The asymmetry between these measurement approaches reflects the fundamentally different sources of uncertainty in human and artificial systems and should be regarded as an inherent feature of the framework rather than a limitation.

**Figure 8:**
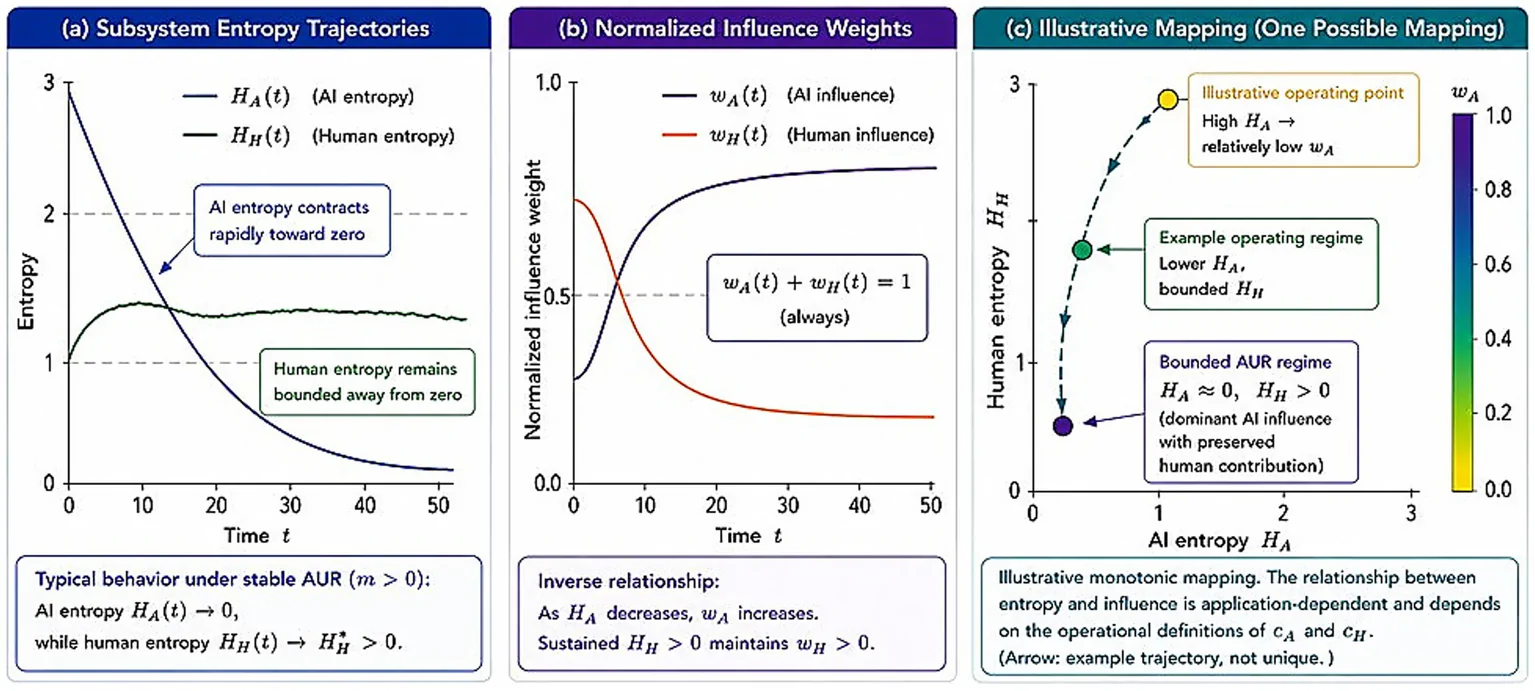
Representative relationship between subsystem entropy and normalized influence weights within the AUR framework. As AI predictive entropy decreases, effective AI influence increases, while bounded human entropy preserves complementary human influence. The mapping is illustrative and application dependent.

Accordingly, Equation (19) should be interpreted as a generic normalization framework rather than as prescribing identical confidence metrics for human and AI subsystems. The specific operationalization of $c_{A}$ and $c_{H}$ should be selected according to the characteristics of the application and the available measurements. A representative mapping between entropy and influence is shown in Figure 8.

This decomposition is a derived observable, not an independent dynamical variable. It establishes an inverse relationship between AI predictive entropy and effective influence while preserving the independent stochastic–affine evolution of $H_{A}(t)$ and $H_{H}(t)$ .

### 5.6 Empirical tests and falsification

A central challenge to the AUR framework is whether asymmetric entropy regulation is genuinely necessary for adaptive resilience, or whether comparable performance can be achieved using existing approaches such as robust control, bounded rationality heuristics, or Bayesian active learning without preserving asymmetric uncertainty. AUR would be theoretically challenged if systems exhibiting progressive symmetric uncertainty suppression, in which both human and AI entropy contract toward minimal exploratory variability, maintained sustained adaptability under severe out-of-distribution conditions.

More directly, AUR would be empirically weakened if maintenance of bounded human entropy $\left(H_{H}^{*}>0\right)$ , preservation of structural information $\left(I_{\text{struct}}\right)$ , or satisfaction of the determinant stability condition (m>0) failed to predict resilience, adaptive flexibility, or recovery under distributional perturbation.

AUR is not proposed as an optimization algorithm intended to replace methods such as Bayesian active learning. Rather, it provides a dynamical state-space framework in which entropy serves as a structural observable for characterizing the stability of coupled human–AI systems through the interaction matrix M and stability margin *m*.

Failure of entropy-based observables to predict decision quality does not, by itself, falsify AUR. Such findings may instead indicate that entropy alone is insufficient to characterize coupled human–AI behavior, particularly when either subsystem relies on biased representations or incorrect models. The framework is falsified only if its structural predictions regarding the coupled entropy dynamics fail, rather than because entropy measures alone cannot explain all aspects of joint task performance (Hemmatian et al., 2024).

An additional empirical challenge is distinguishing adaptive from maladaptive preserved uncertainty. AUR predicts that bounded human entropy and structural divergence should improve resilience, contextual adaptation, and recovery under distributional shift. Conversely, if elevated entropy or structural divergence consistently correlate with poorer decision quality, increased bias, or degraded adaptation, this interpretation would be weakened.

A further limitation concerns the optimal magnitude of stationary human entropy. Although the framework predicts that $\left(H_{H}^{*}>0\right)$ is necessary to preserve exploratory flexibility, it does not specify the task-dependent optimum. Insufficient residual entropy may produce premature convergence and overreliance on AI recommendations, whereas excessive entropy may impair coherence, calibration, or decision quality. Determining the appropriate degree of asymmetry therefore requires empirical evaluation using external performance measures, including accuracy, resilience under distributional shift, adaptation to novelty, and decision quality. Future work should relate AUR to established exploration–exploitation frameworks in reinforcement learning, multi-armed bandits, and collective adaptation in behavioral ecology and cultural evolution (Boyd and Richerson, 2005).

Controlled benchmarking studies comparing AUR-informed human–AI systems with conventional architectures under strict out-of-distribution conditions may further determine whether structural information (I_struct_) functions as a predictive marker of resilience, adaptability, and sustained performance.

### 5.7 Computational reproducibility

The illustrative figures and entropy trajectories presented in this work are derived directly from the governing equations and representative parameter values reported throughout the manuscript. The figures are conceptual illustrations intended to visualize the qualitative dynamical regimes implied by the analytical properties of the AUR framework rather than empirically calibrated simulations or stochastic Monte Carlo experiments.

Unless otherwise stated, deterministic trajectories correspond to the drift dynamics of Equation 4, while the illustrative stochastic behavior reflects the phenomenological formulation introduced in Section 3. The representative parameter values used in the domain-specific examples are reported in the corresponding sections and tables.

The analytical formulation presented in this manuscript is fully specified by the governing equations and parameter values. Researchers wishing to reproduce or extend the illustrative examples may implement the model directly from these equations using numerical methods of their choice.

## 6 Normative implications of entropy dynamics

The AUR framework is not intended as a universal theory of historical or societal progress, but as a dynamical framework for analyzing stability, adaptability, and uncertainty regulation in coupled human–AI systems. The stochastic–affine formulation developed in Sections 3 and 4 carries several normative implications for the design and governance of such systems.

### 6.1 Autonomy as non-vanishing entropy

Within AUR, human autonomy is interpreted as a structural dynamical property rather than a psychological state: the preservation of non-vanishing stationary human entropy $\left(H_{H}^{*}>0\right)$ . If the exogenous entropy injection η̄ vanishes while λ_H > 0 remains positive, the stationary solution collapses to $H_{H}^{*}\;$ = 0, reducing the human subsystem to a deterministic extension of the AI system and eliminating the capacity for independent reinterpretation, preference revision, and counterfactual reasoning. This interpretation is consistent with philosophical accounts of agency emphasizing reflective endorsement and deliberative capacity (Frankfurt, 1971; Dennett, 1984).

Preserving autonomy therefore requires maintaining sufficient entropy slack to support genuine human agency within the coupled system.

### 6.2 Responsibility and entropic asymmetry

Meaningful responsibility likewise depends on preserving entropic asymmetry. To endorse, revise, or reject AI recommendations in a non-trivial manner, the human subsystem must retain bounded uncertainty while AI uncertainty contracts more rapidly. Symmetric uncertainty minimization (λ_A_ ≈ λ_H_, η̄ → 0) removes this asymmetry by driving both subsystems toward a common deterministic trajectory, thereby undermining meaningful human oversight.

Responsibility is therefore not only a legal or social concept, but also a dynamical property of the coupled system.

### 6.3 Ethical design principles

The entropy-dynamical analysis suggests four principles for ethically robust human–AI systems, broadly consistent with human-centered AI approaches emphasizing reliability, human oversight, and preserved human agency (Shneiderman, 2020):

- *Maintain appropriate asymmetry*: AI uncertainty should contract sufficiently faster than human uncertainty to preserve meaningful human oversight. The appropriate degree of asymmetry is task-dependent and should balance rapid AI inference with the human operator’s ability to interpret, verify, and challenge AI recommendations.
- *Safeguard human exploratory capacity*: Preserve positive exogenous entropy injection (η̄ > 0) by supporting contextual reasoning, preference evolution, and creative reinterpretation.
- *Maintain structural stability*: Ensure the determinant condition λ_A_ λ_H_ > γδ (or equivalently, *m* > 0) so that cross-subsystem coupling remains stable.
- *Avoid symmetric uncertainty collapse*: Designs that drive both entropies toward zero may be efficient in stationary environments but become brittle under distributional shift.

AUR should not be interpreted as an alternative theory of trust, automation bias, or algorithm acceptance. Rather, it provides a complementary dynamical perspective in which automation bias may correspond to excessive contraction of human exploratory entropy, algorithm aversion to insufficient utilization of AI predictive contraction, and calibrated trust to operation within the stable asymmetric regime. From this perspective, these behavioral phenomena can be interpreted as different regions of the coupled uncertainty state space rather than independent mechanisms.

### 6.4 Robustness and boundary conditions

In stationary environments, global entropy minimization may appear optimal. Under structural uncertainty and distributional shift, however, bounded human entropy provides an essential exploration buffer. AUR occupies the intermediate regime in which predictive accuracy is enhanced while adaptive capacity is preserved. Maintaining the system within the stable regime identified in Section 4 is therefore both a mathematical requirement and a practical condition for resilient and ethically robust human–AI collaboration.

## 7 Conclusion

The Asymmetric Uncertainty Regulation framework reconciles two seemingly opposing goals: the drive toward predictive optimization on one hand, and the preservation of human autonomy and responsibility on the other. By showing that these goals are best achieved through asymmetric rather than uniform regulation of uncertainty, AUR provides both a mathematical structure and a normative design principle for human–AI systems. The framework is intended to complement existing research on human-centered AI, trust calibration, automation bias, and complementary decision-making by providing a common entropy-dynamical language for analyzing coupled human–AI interactions.

Taken together with our prior work on free will as *structured unpredictability*, the present framework offers a unified view: human agency is not an obstacle to be minimized, but a structural feature to be deliberately preserved within stable dynamical bounds. Effective and ethically sound human–AI collaboration therefore does not require the elimination of uncertainty. It requires its disciplined, asymmetric regulation, rapid contraction in the predictive (AI) domain and bounded preservation in the human domain.

This principle suggests potential implications for the design, evaluation, and governance of human–AI systems, subject to future empirical investigation. Future work should test the empirical predictions of AUR across diverse domains, refine the measurement of entropy trajectories, and explore extensions to multi-agent and multi-modal human–AI systems. If supported by future empirical studies, the long-term success of human–AI collaboration may depend less on maximizing AI autonomy alone and more on designing systems that preserve and augment human autonomy within stable asymmetric architectures.

## Data Availability

The original contributions presented in the study are included in the article/Supplementary material, further inquiries can be directed to the corresponding author.
