# Supplementary material for: Asymmetric uncertainty regulation in human–artificial intelligence interaction
Source: Front Artif Intell. 2026 Sep 1;9:1847409. doi: 10.3389/frai.2026.1847409 (PMC13574801; doi:10.3389/frai.2026.1847409)
Supplement: Supplementary file 1 [file Data_Sheet_1.PDF]

Supplementary Material

Asymmetric Uncertainty Regulation in Human–Artificial Intelligence Interaction

Abbaspour et al.

Illustrative Case Studies

To demonstrate the operational meaning of Asymmetric Uncertainty Regulation (AUR) across domains, we present three case studies that instantiate the same coupled entropy framework under different temporal realizations and stress conditions. The first example (Nutritional Counseling), adopts a discrete-time, session-based representation appropriate for episodic human–AI interaction. In this example, AUR predicts a regime in which AI uncertainty contracts rapidly while human uncertainty remains bounded yet non-vanishing, preserving adaptive flexibility without sacrificing system stability. Breakdown of this asymmetric structure yields either symmetric collapse (premature convergence) or instability (loss of bounded regulation), providing empirically testable signatures across domains.

It should be noted that in the examples provided below the numerical parameter values used are illustrative and chosen only to demonstrate the qualitative behavior of the AUR dynamics (rapid AI entropy contraction and slower human entropy relaxation). The conclusions do not depend on these specific values but on the inequality $\lambda_A \lambda_H > \gamma\delta$, which defines the stability regime.

In empirical applications, these parameters could be estimated from observed decision dynamics, such as AI model confidence convergence rates, human revision frequency, or investigative hypothesis turnover.

Example I: Nutritional Counseling

Appendix A. Detailed Case Study: Nutritional Counseling

A1. Conceptual Framework and Observables

Personalized nutritional counseling provides a transparent domain for illustrating the mechanics of Asymmetric Uncertainty Regulation (AUR). Dietary intervention selection requires balancing population-level clinical evidence with individualized patient factors, including cultural habits, daily routines, adherence feasibility, motivational variability, and evolving preferences.

At each counseling session t, both the AI model and the practitioner maintain probability distributions over a discrete intervention space consisting of four candidate dietary plans: Mediterranean, lower-carbohydrate, plant-forward, and DASH-like:

$$P_A(t) = (p_{\text{Med}}(t), p_{\text{LC}}(t), p_{\text{PF}}(t), p_{\text{DASH}}(t))$$

$$P_H(t) = (q_{\text{Med}}(t), q_{\text{LC}}(t), q_{\text{PF}}(t), q_{\text{DASH}}(t))$$

The uncertainty of each subsystem is quantified using Shannon entropy:

$$H_A(t) = -\sum_{j=1}^{4} p_j\,(t)\ln\ p_j(t)$$
$$H_H(t) = -\sum_{j=1}^{4} q_j\,(t)\ln\ q_j(t)$$

These values define the coupled entropy state vector:

$$\mathbf{H}(t) = \begin{pmatrix} H_A(t) \\ H_H(t) \end{pmatrix}$$

A2. Parametric Setup and Stability Verification

The coupled system is modeled using the continuous-time stochastic affine equation:

$$d\mathbf{H}(t) = \big(M\mathbf{H}(t) + \bar{\mathbf{b}}\big)dt + G(\mathbf{H}(t))dW(t)$$

For the nutritional counseling example, we use the following stable AUR parameterization:

$$M = \begin{pmatrix} -\lambda_A & \gamma \\ \delta & -\lambda_H \end{pmatrix} = \begin{pmatrix} -1.20 & 0.10 \\ 0.02 & -0.15 \end{pmatrix}, \bar{\mathbf{b}} = \begin{pmatrix} b_A \\ \bar{\eta} \end{pmatrix} = \begin{pmatrix} 0.00 \\ 0.205 \end{pmatrix}$$

This configuration satisfies the AUR conditions. First, the AI subsystem contracts uncertainty faster than the human subsystem:

$$\lambda_A = 1.20 \gg \lambda_H = 0.15$$

Second, the trace is negative:

$$\text{tr}(M) = -(\lambda_A + \lambda_H) = -(1.20 + 0.15) = -1.35 < 0$$

Third, the determinant stability margin is positive:

$$m = \lambda_A\lambda_H - \gamma\delta$$
$$m = (1.20)(0.15) - (0.10)(0.02) = 0.180 - 0.002 = 0.178 > 0$$

Thus, the system lies inside the stable AUR regime.

A3. Microstate-Level Entropy Trajectories

To show how the entropy trajectories arise from explicit probability distributions, we provide three observation windows.

Session 0: Initial Assessment

$$P_A(0) = (0.40, 0.30, 0.20, 0.10)$$
$$H_A(0) = 1.28$$
$$P_H(0) = (0.20, 0.35, 0.25, 0.20)$$
$$H_H(0) = 1.36$$

The structural information component is:

$$I_{\text{struct}}(0) = D_{KL}(P_H(0) \parallel P_A(0)) = 0.42$$

At the initial assessment, both the AI and practitioner retain substantial uncertainty. The AI distribution reflects population-level uncertainty across candidate interventions, while the practitioner distribution reflects broader contextual consideration of patient-specific factors.

Session 3: Midpoint Accumulation

$$P_A(3) = (0.76, 0.12, 0.10, 0.02)$$
$$H_A(3) = 0.77$$
$$P_H(3) = (0.15, 0.40, 0.35, 0.10)$$
$$H_H(3) = 1.37$$
$$I_{\text{struct}}(3) = D_{KL}(P_H(3) \parallel P_A(3)) = 0.78$$

By the midpoint, accumulated biomarker and dietary-response information allows the AI to concentrate probability mass on a narrower statistical recommendation. The practitioner, however, maintains a broader distribution because contextual considerations remain active, including patient adherence, meal routines, food access, and motivational readiness.

Session 7: Asymmetric Operating Regime

$$P_A(7) = (0.91, 0.05, 0.03, 0.01)$$
$$H_A(7) = 0.39$$
$$P_H(7) = (0.10, 0.45, 0.40, 0.05)$$
$$H_H(7) = 1.37$$
$$I_{\text{struct}}(7) = D_{KL}(P_H(7) \parallel P_A(7)) = 1.12$$

By Session 7, the AI subsystem has strongly contracted predictive entropy, whereas the practitioner retains bounded non-zero entropy. The increasing structural divergence is consistent with maintained context-sensitive judgment rather than simple convergence toward the algorithmic recommendation.

A4. Stationary Mean Calculation

The deterministic mean trajectory converges toward the stationary operating point:

$$\mathbf{H}^* = -M^{-1}\bar{\mathbf{b}}$$

The inverse matrix is:

$$M^{-1} = \frac{1}{\det(M)}\begin{pmatrix} -\lambda_H & -\gamma \\ -\delta & -\lambda_A \end{pmatrix} = \frac{1}{0.178}\begin{pmatrix} -0.15 & -0.10 \\ -0.02 & -1.20 \end{pmatrix}$$

Therefore:

$$\mathbf{H}^* = -\frac{1}{0.178}\begin{pmatrix} -0.15 & -0.10 \\ -0.02 & -1.20 \end{pmatrix}\begin{pmatrix} 0 \\ 0.205 \end{pmatrix}$$

$$\mathbf{H}^* = \frac{1}{0.178}\begin{pmatrix} 0.10 \times 0.205 \\ 1.20 \times 0.205 \end{pmatrix}$$

$$H_A^* = \frac{0.0205}{0.178} \approx 0.115$$

$$H_H^* = \frac{0.246}{0.178} \approx 1.382$$

This calculation demonstrates that, under the specified AUR parameterization, the system converges toward a strongly contracted AI entropy state while maintaining a persistent positive human entropy plateau.

A5. Domain Interpretation

In this example, the AI subsystem progressively contracts predictive uncertainty as patient-specific evidence accumulates. This reflects its role as a statistical optimization engine that increasingly concentrates probability mass around the intervention most supported by available data.

The practitioner subsystem does not mirror this contraction. Instead, human entropy remains bounded near a positive operating level. This residual uncertainty is not interpreted as random indecision, but as preserved contextual flexibility sustained by continuous patient-specific information input $\bar{\eta} = 0.205$. Such input represents factors that are difficult to fully encode in the AI model, including cultural preference, adherence likelihood, emotional readiness, household constraints, and changing patient goals.

The structural information component,

$$I_{\text{struct}} = D_{KL}(P_H \parallel P_A),$$

increases across the observation windows:

$$0.42 \rightarrow 0.78 \rightarrow 1.12$$

This increasing divergence is consistent with the practitioner maintaining a structured, context-sensitive interpretive space rather than simply collapsing toward the AI recommendation. Within the AUR framework, this pattern illustrates how predictive optimization and human contextual judgment can coexist: the AI narrows statistical uncertainty, while the practitioner preserves bounded exploratory variability necessary for individualized care.

6. The Patient Takeaway: What AUR Looks Like in Practice

From the patient's perspective, an interface built on the principles of Asymmetric Uncertainty Regulation avoids the two primary failure modes of modern medical AI deployment: **algorithmic prescription** (where the clinician blindly reads off a computer screen) and **unconstrained improvisation** (where clinical data is ignored in favor of pure guesswork).

Instead, the patient experiences a deeply personalized, scientifically backed care pathway:

- **Session 0 (The Discovery Phase):** The patient receives a comprehensive biochemical and lifestyle intake. The AI aggregates thousands of data points to propose a wide range of clinically viable pathways (Mediterranean, low-carbohydrate, plant-forward), keeping options open while the human practitioner actively interviews the patient to gauge their underlying motivations, cultural food preferences, and daily stresses.
- **Session 3 (The Convergence Phase):** As biomarker tracking continues, the AI rapidly identifies the mathematically optimal foundation—for instance, showing that a Mediterranean framework yields the highest statistical probability of improving the patient's specific metabolic markers. The system narrows down, giving the patient clear, data-backed direction.
- **Session 7 (The Personalized Stabilization Phase):** At this stage, a non-AUR system would hand down a rigid, automated meal plan. Under the AUR framework, however, the practitioner uses their preserved uncertainty plateau to contextualize the AI's core recommendation. The patient does not leave with a

cold, computer-generated mandate; instead, they leave with a *"Mediterranean-dominant framework"* that has been actively modified by the clinician to accommodate the patient's unique work schedule, family dynamics, and budget constraints.

Ultimately, AUR ensures that the machine is utilized for what it does best—rapidly crunching statistics and minimizing data uncertainty—while preserving the human practitioner's cognitive space to deliver empathetic, flexible, and context-aware healthcare.
